# Structural remodeling and conduction velocity dynamics in the human left atrium: Relationship with reentrant mechanisms sustaining atrial fibrillation

**DOI:** 10.1016/j.hrthm.2018.07.019

**Published:** 2019-01

**Authors:** Shohreh Honarbakhsh, Richard J. Schilling, Michele Orini, Rui Providencia, Emily Keating, Malcolm Finlay, Simon Sporton, Anthony Chow, Mark J. Earley, Pier D. Lambiase, Ross J. Hunter

**Affiliations:** Barts Heart Centre, Barts Health NHS Trust, London, United Kingdom

**Keywords:** Atrial fibrillation, Bipolar voltage, Conduction velocity, Drivers, Structural remodeling

## Abstract

**Background:**

Rate-dependent conduction velocity (CV) slowing is associated with atrial fibrillation (AF) initiation and reentrant mechanisms.

**Objective:**

The purpose of this study was to assess the relationship between bipolar voltage, CV dynamics, and AF drivers.

**Methods:**

Patients undergoing catheter ablation for persistent AF (<24 months) were enrolled. Unipolar electrograms were recorded with a 64-pole basket catheter during atrial pacing at 4 pacing intervals (PIs) during sinus rhythm. CVs were measured between pole pairs along the wavefront path and correlated with underlying bipolar voltage. CV dynamics within low-voltage zones (LVZs <0.5 mV) were compared to those of non-LVZs (≥0.5 mV) and were correlated to driver sites mapped using CARTOFINDER (Biosense Webster).

**Results:**

Eighteen patients were included (age 62 ± 10 years). Mean CV at 600 ms was 1.59 ± 0.13 m/s in non-LVZs vs 0.98 ± 0.23 m/s in LVZs (*P* <.001). CV decreased incrementally over all 4 PIs in LVZs, whereas in non-LVZs a substantial decrease in CV was only seen between PIs 300–250 ms (0.59 ± 0.09 m/s; *P* <.001). Rate-dependent CV slowing sites measurements, defined as exhibiting CV reduction ≥20% more than the mean CV reduction seen between PIs 600–250 ms for that voltage zone, were predominantly in LVZs (0.2–0.5 mV; 75.6% ± 15.5%; *P* <.001). Confirmed rotational drivers were mapped to these sites in 94.1% of cases (sensitivity 94.1%, 95% CI 71.3%–99.9%; specificity 77.9%, 95% CI 74.9%–80.7%).

**Conclusion:**

CV dynamics are determined largely by the extent of remodeling. Rate-dependent CV slowing sites are predominantly confined to LVZs (0.2–0.5 mV), and the resultant CV heterogeneity may promote driver formation in AF.

## Introduction

The effect of structural remodeling on human left atrial (LA) conduction velocity (CV) has been studied previously.^1^ Low-voltage zones (LVZs) defined by bipolar voltage^1^ and late gadolinium enhancement on cardiac magnetic resonance imaging^2^ have demonstrated lower local CVs, which may promote reentry. This forms the rationale for current strategies ablating LVZs for atrial fibrillation (AF).^2^, ^3^

A direct relationship between bipolar voltage and CV in the human LA has not been established. Therefore, how CV changes over the spectrum of structural remodeling seen in AF is unclear. CV dynamic curves in myocardial tissue demonstrate a steep CV reduction with a decrease in pacing interval (PI).^4^ In the presence of structural remodeling, these curves are shifted to the right, whereby the rate-adapted CV slowing occurs at longer PIs.^5^ These differences in CV dynamics have been shown to contribute to reentry.^4^ Sites with marked slowing of CV with increasing rate (ie, rate-dependent CV slowing) have been shown to be associated with the ability to induce AF^5^ and correspond to sites of reentry initiation at AF onset.^6^

We aimed to determine the interaction between remodeling and CV dynamics by analyzing LVZs and local CVs in the human LA using a 64-pole basket catheter. CV dynamics were compared across a range of voltages, and the presence of rate-dependent CV slowing was evaluated. The CARTOFINDER mapping system (Biosense Webster, Diamond Bar, CA) was used to map AF^7^ to assess whether abnormalities in CV dynamics are directly linked to the mechanisms sustaining AF.

## Method

### Study design

Patients undergoing catheter ablation for persistent AF (<24 months and no previous ablation) and in sinus rhythm at the start of the case (before direct current cardioversion) were included. Patients provided informed consent, and the study was approved by the UK Research Ethics Committee (London-Bloomsbury Research Ethics Committee, 16/LO/1379).

### Electrophysiological mapping

Mapping was performed using the CARTOFINDER system (see Supplemental Methods).^7^

LA geometry and high-density bipolar voltage map were created using a PentaRay NAV catheter with 2-6-2 mm electrode spacing (Biosense Webster) (see Supplemental Methods). Voltage zones were defined as non-LVZ (≥0.5 mV), LVZ (0.2–0.5 mV), and very LVZ (vLVZ) (0–0.2 mV).^8^ Unipolar electrograms were obtained using a LabSystem Pro electrophysiological recording system (Bard Electrophysiology Division, Natick, MA) by referencing to a decapolar catheter (Biosense Webster) positioned in the inferior vena cava. Filter bandwidth was 0.05–500 Hz.

A 64-pole basket catheter (Constellation, Boston Scientific Ltd, Natick, MA; or FIRMap, Abbott, Santa Clara, CA) was used to record unipolar signals. It was positioned in the LA to achieve the best possible atrial coverage.^9^

### Pacing procedure

To achieve wavefront propagations in different directions, atrial pacing was performed in sinus rhythm with the ablation catheter from 4 sites: proximal and distal coronary sinus (endocardial), LA roof, and LA appendage. Uninterrupted pacing was performed at 4 PIs: 600, 450, 300, and 250 ms. For each PI, 30-seconds of unipolar electrograms were recorded at a sampling frequency of 2000 Hz. A location point was also taken on CARTO to obtain 3-dimensional coordinates for each pole.

### Local CVs

CV was defined as the geodesic distance divided by the activation time difference and expressed in m/s (see Supplemental Methods).^10^

### CV dynamics and bipolar voltage

Using a MATLAB custom-written script (The MathWorks, Natick, MA), the position of the electrode pairs included in the analysis and bipolar voltage points taken with the PentaRay catheter were projected onto the LA geometry. The bipolar voltage points were considered within a 5-mm band between the electrodes from which CV was assessed. The mean of these was taken as the local bipolar voltage along the path between each electrode pair and used to define the voltage zone along the path.

When the distance between electrodes for measurement of CV was seen to traverse different voltage zones, then measurements were excluded so as to avoid CV measurements over heterogenous tissues. CV at each PI was compared in the 3 voltage zones. To evaluate for heterogeneity in CV dynamics within LVZs and non-LVZs, sites of rate-dependent CV slowing were identified. These were defined as zones exhibiting a reduction in CV between PI = 600 ms and PI = 250 ms of ≥20% more than the mean CV reduction seen between these PIs for that voltage zone.

### CV and AF driver sites

AF was induced after the study protocol by burst atrial pacing from the coronary sinus (see Supplemental Methods).

CARTOFINDER maps were then created before and after pulmonary vein isolation (PVI), and the maps post-PVI were used to guide further ablation. A potential driver was defined as either (1) focal with radial activation over ≥2 consecutive wavefronts or (2) rotational activity with ≥1.5 rotations of 360°.^7^ Confirmed drivers were defined as sites at which ablation terminated AF into atrial tachycardia (AT) or sinus rhythm or slowed AF cycle length by ≥30 ms.^7^

### Ablation strategy

The ablation strategy used in this study has previously been defined (see Supplemental Methods).^7^

### Statistical analysis

Statistical analysis was performed using SPSS Statistics Version 24 (IBM Corp, Armonk, NY). Continuous variables are given as mean ± SD. Categorical variables are given as number (percentage). The χ^2^ test was used for comparison of nominal variables. The Student t-test or its nonparametric equivalent, the Mann-Whitney *U* test, was used when appropriate for comparison of continuous variables. Spearman rank order correlation was used to assess the strength and association between CV and bipolar voltage. The paired Student *t* test was used to compare CVs obtained for each PI. One-way analysis of variance was used to compare CV changes over the PIs between voltage zones. Receiver operating characteristic curves were obtained to determine the diagnostic ability of different parameters in predicting driver sites. *P* <.05 was considered significant.

## Results

Eighteen patients were included in the study (Supplemental Table 1).

### Bipolar voltage and CV

**Figure 1:**
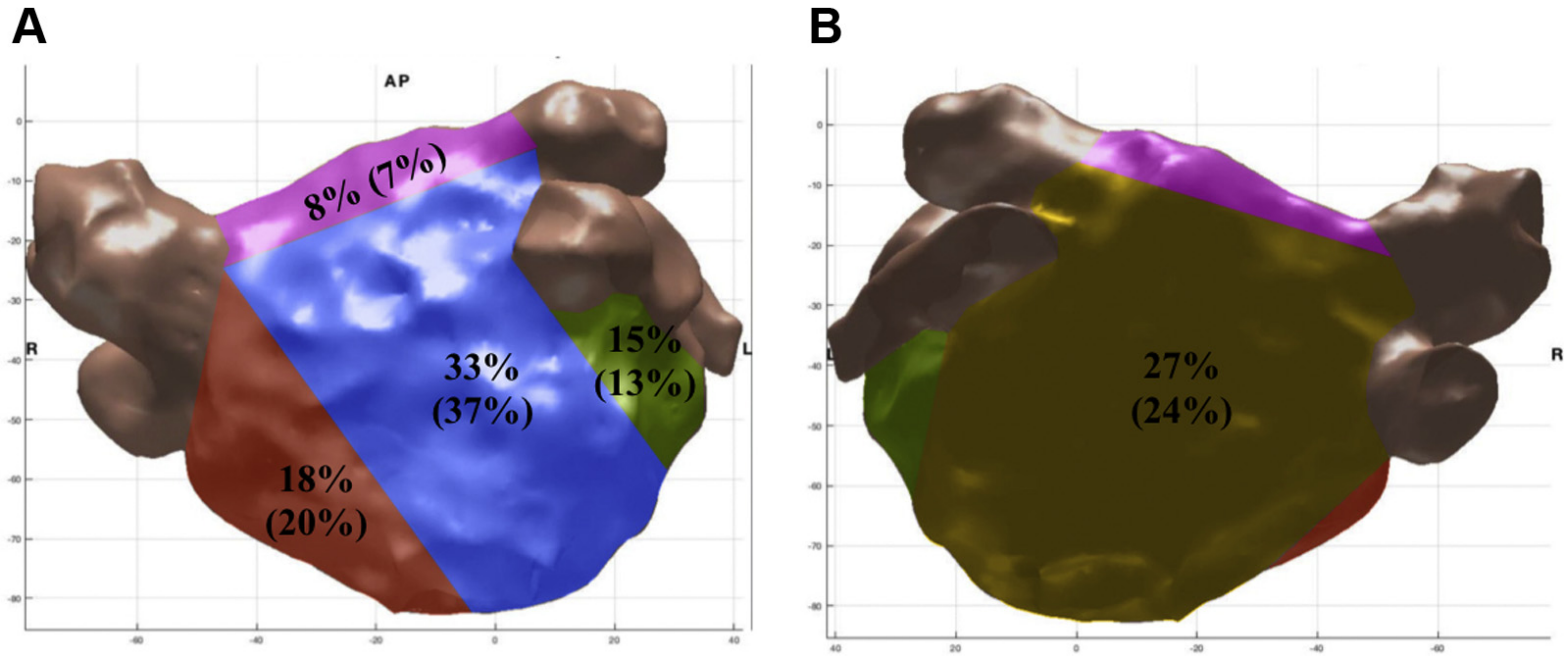
Anterior (**A**) and posterior (**B**) views of the left atrium showing the distribution of the low-voltage zones and rate-dependent conduction velocity slowing sites (percentage in brackets) in the patients involved in the study. *Red* indicates septum; *green* indicates lateral; *blue* indicates anterior; *yellow* indicates posterior; *purple* indicates roof.

A total of 15,363 bipolar voltage points were taken with an average of 854 ± 240 points per patient, of which 487 ± 188 points were <0.5 mV (57% ± 22%). Mean bipolar voltage was 0.43 ± 0.18 mV. LVZs were found to occur as islands or plaques, each one covering a minimum of 10% of the LA surface (29% ± 15%). LVZs predominantly affected the anterior wall (33%) (Figure 1).

**Figure 2:**
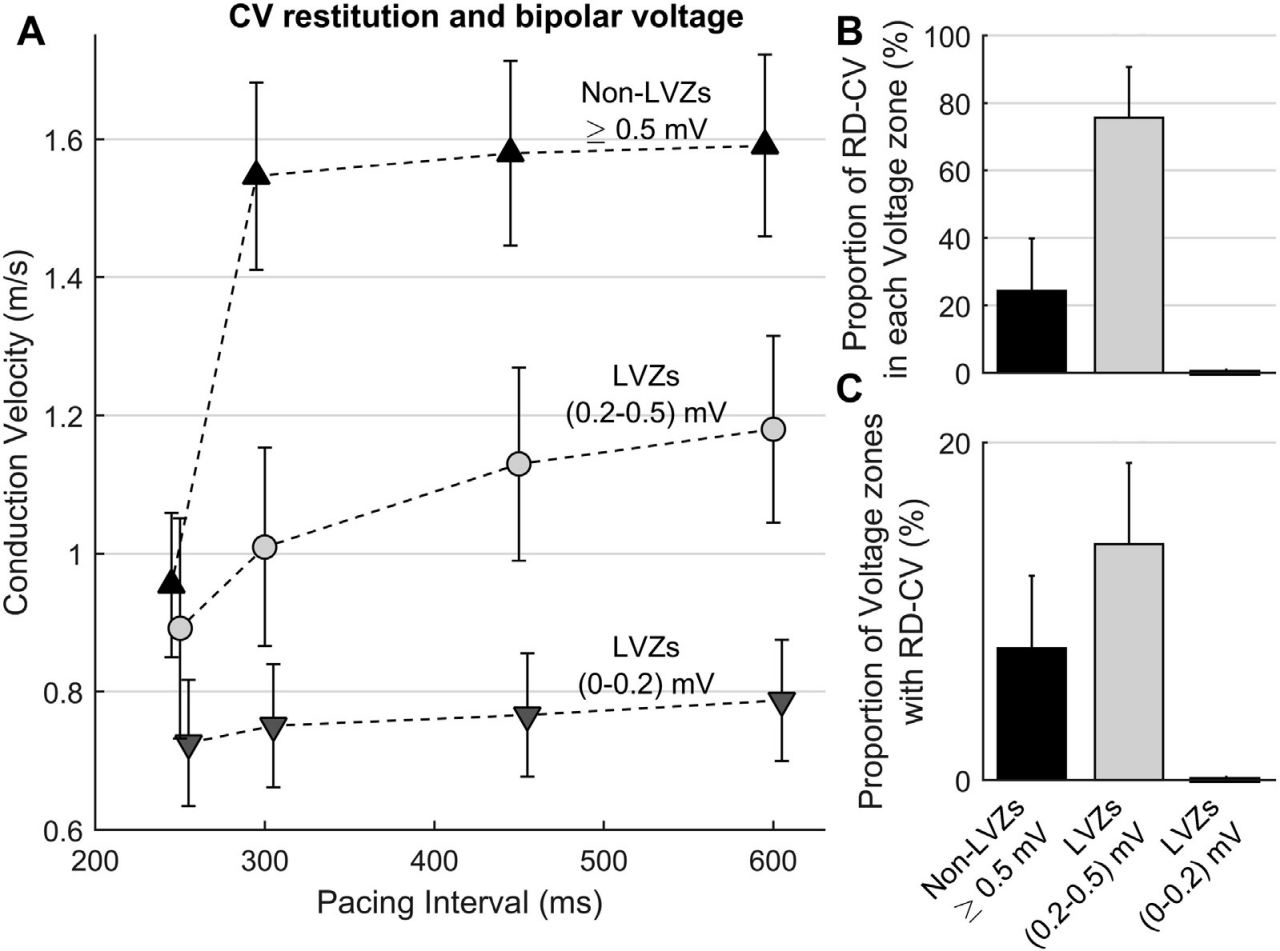
**A:** Change in CV over the 4 pacing intervals in non-LVZs ≥0.5 mV (*black triangles*), LVZs (0.2–0.5 mV) (*light gray circles*), and LVZs (0–0.2 mV) (*dark gray triangles*). **B, C:** Bar chart showing the percentage of rate-dependent CV slowing sites in non-LVZs ≥0.5 mV, LVZs (0.2–0.5 mV), and LVZs (0–0.2 mV) (**B**) and the proportion of non-LVZs ≥0.5 mV, LVZs (0.2–0.5 mV), and LVZs (0–0.2 mV) demonstrating rate-dependent CV slowing (**C**). CV = conduction velocity; LVZ = low-voltage zone; RD-CV = rate-dependent conduction velocity.

**Figure 3:**
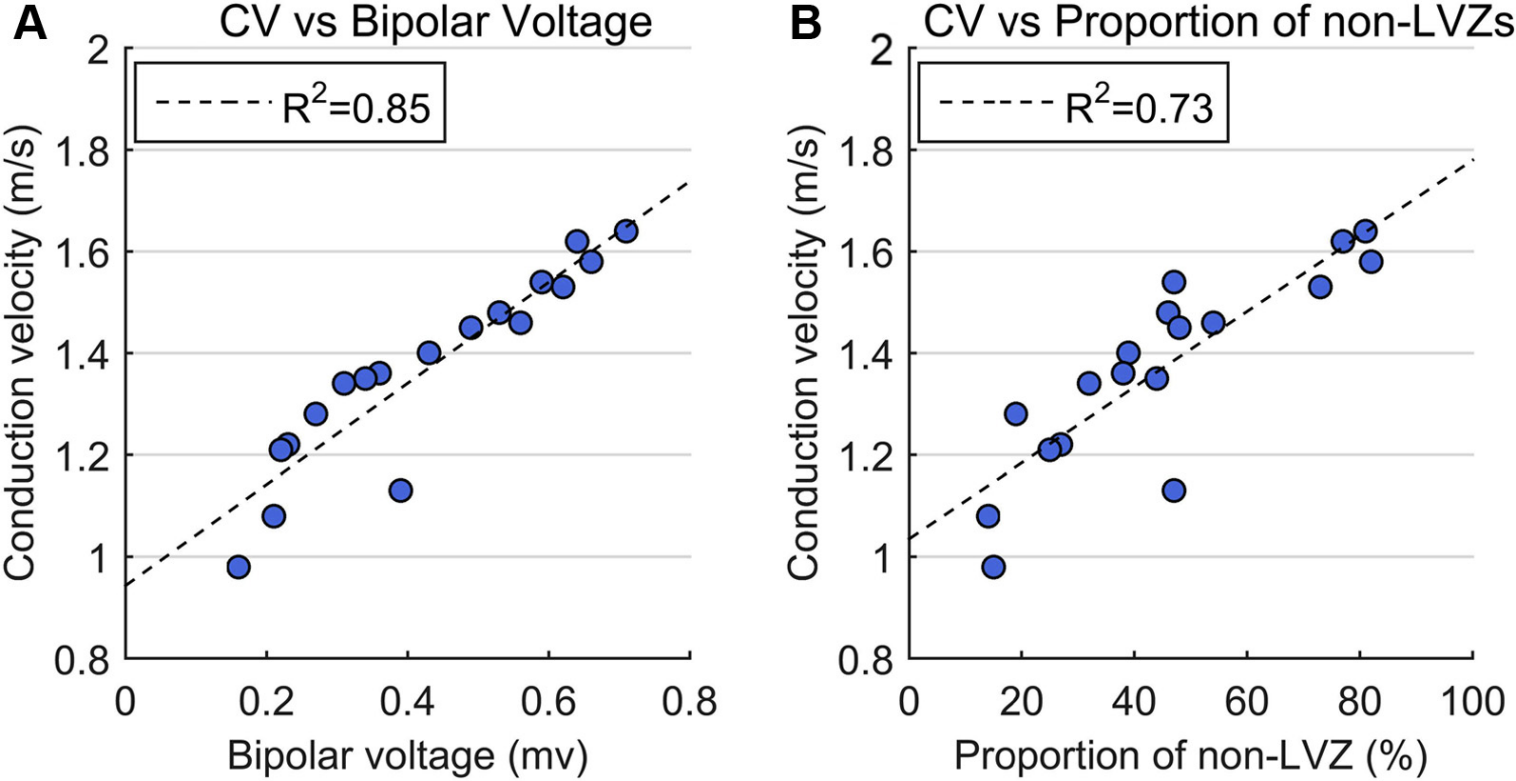
Relationship between mean CV for each patient at pacing interval of 600 ms (the average of all the CV measured between all pole pairs in each patient) and mean bipolar voltage including all bipolar voltage points in each patient (**A**) and the proportion of non-LVZs in each patient (**B**). CV = conduction velocity; LVZ = low-voltage zone.

CV was determined over a total of 3197 electrode pairs, with a mean of 45.8 ± 8.2 pairs for each activation sequence in each patient. At PI = 600 ms, mean CV was higher in non-LVZs than LVZs (Figure 2A). There was a positive correlation between the mean CV for a patient and both mean bipolar voltage (r_s_ = 0.95; *P* <.001) (Figure 3A) and proportion of non-LVZs (r_s_ = 0.87; *P* <.001) (Figure 3B).

### CV dynamics and bipolar voltage

As shown in Figure 2A and Supplemental Table 2, CV dynamics significantly differed at sites of non-LVZs, LVZs (0.2–0.5 mV) and vLVZs (0–0.2 mV). In non-LVZs, CVs only significantly changed between PIs of 300–250 ms (0.59 ± 0.09 m/s; *P* <.001), with a total average reduction of 62.9% ± 6.9% between PIs 600–250 ms, whereas the activation time increased by an average of 12 ± 5 ms. In LVZs (0.2–0.5 mV), there was a progressive decrease in CV between all 4 PIs, with a total average reduction of 23.7% ± 13.6% between PIs 600–250 ms. The activation time increased by an average of 9 ± 3 ms. In vLVZs (0–0.2 mV), there was minimal change in CV with increased pacing rate (mean change 0.02 ± 0.02 m/s over each PI; *P* =.48).

### Relationship between rate-dependent CV slowing sites and bipolar voltage

For each pacing site, a mean of 10.7 ± 4.3 rate-dependent CV slowing measurements were observed per patient (24.0% ± 11.9% of sites sampled), with a mean 1.46 ± 0.64 rate-dependent CV slowing sites per patient (total of 41 rate-dependent CV slowing sites in the 18 patients), which were spatially stable. Rate-dependent CV slowing site measurements were significantly more common in LVZs than non-LVZs (75.6% ± 15.5% vs 24.4% ± 15.0; *P* <.001) (Figure 2B). In all, 14.0% ± 4.3% of LVZs exhibited rate-dependent CV slowing compared to 7.8% ± 4.8% of non-LVZs (*P* <.001). Rate-dependent CV slowing sites were more prevalent in patients with a lower mean bipolar voltage (r_s_ = –0.91; *P* <.001). Rate-dependent CV slowing sites were more commonly mapped to the anterior (37%) and posterior (24%) wall, which correlated to sites where LVZs were more frequent (Figure 1).

Rate-dependent CV slowing sites in LVZs were all LVZs (0.2–0.5 mV) and showed progressive decrease in CV over all 4 PIs (mean decrease in CV of 0.12 ± 0.03 m/s for each PI), resulting in broader curves. Rate-dependent CV slowing sites in non-LVZs showed the greatest decrease in CV between PIs of 300–250 ms (mean decrease in CV of 0.70 ± 0.09 m/s; *P* = .001), with minimal change at longer PIs, resulting in a steeper curve.

### Relationship between LVZs and rate-dependent CV slowing and drivers

In the 18 AF patients, 29 possible drivers were identified using the CARTOFINDER system (1.6 ± 0.7 drivers per patient; n = 18 rotational activity and n = 11 focal activity). Ablation at 25 of the 29 driver sites (n = 8 focal and n = 17 rotational; 86.2%) resulted in an effect that met the study criteria of a confirmed driver (Table 1). With 4 drivers, the predefined ablation response was not seen on ablation (n = 3 focal and n = 1 rotational).

**Table 1:** Mechanism of possible drivers mapped in AF and ablation response

AF drivers	29
Focal activity	11
Rotational activity	18
AF response to ablation at driver site	29
Termination to sinus rhythm	6
Organized to an AT	12
Cavotricuspid isthmus-dependent flutter	2
Mitral isthmus-dependent flutter	4
Roof-dependent flutter	5
Ligament of Marshall	1
Cycle length slowing ≥30 ms	7
No effect of ablation	4

Values are given as n.

AF = atrial fibrillation; AT = atrial tachycardia.

The drivers demonstrated spatial stability but temporal periodicity, with a consecutive repetition of 3.8 ± 1.1 and a recurrence rate of 8.2 ± 4.8 times per 30-second recording.

Eighteen of the 25 confirmed AF drivers were mapped to LVZs (72.0%), which included 15 of the 17 confirmed drivers with rotational activation (88.2%) but only 3 of 8 confirmed drivers with focal activation (37.5%; *P* = .02). Mean bipolar voltage was 0.33 ± 0.10 mV at the confirmed driver sites. No drivers were identified in vLVZs.

**Figure 4:**
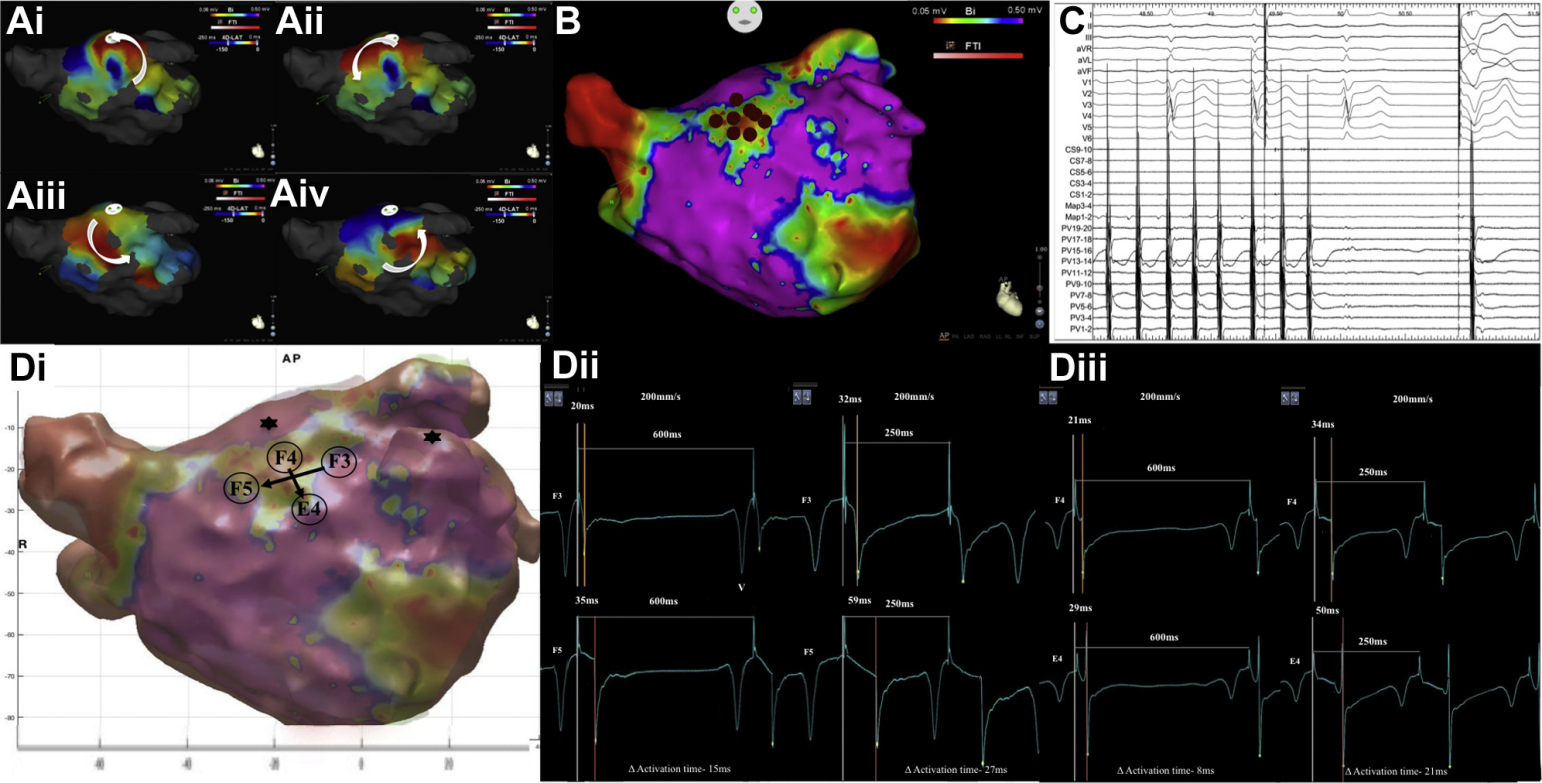
**A: i–iv:** Still CARTOFINDER map demonstrating a rotational driver at the anterior roof (**B**) in an area of LVZ as shown on the bipolar voltage map (**C**), where ablation resulted in atrial fibrillation termination to sinus rhythm on the Bard electrograms. **D: i:** Replicated CARTO geometry created in MATLAB demonstrating site of rate-dependent conduction velocity (CV) slowing at the anterior roof in an area of LVZ (0.2–0.5 mV) (F3–F5 electrodes; vertical and F4–E4 electrode: horizontal). **ii:** Electrograms obtained at F3 and F5 electrodes during left atrial appendage pacing at PI 600–250 ms show an increase in activation time difference of 12 ms (80% increase) between the 2 electrodes when reaching PI of 250 ms. (**iii**) Electrograms obtained at F4 and E4 electrodes during roof pacing at PI 600–250 ms show an increase in activation time difference of 13 ms (163% increase). LVZ = low-voltage zone; PI = pacing interval; V = far-field ventricular signal.

**Supplemental Figure 1:**
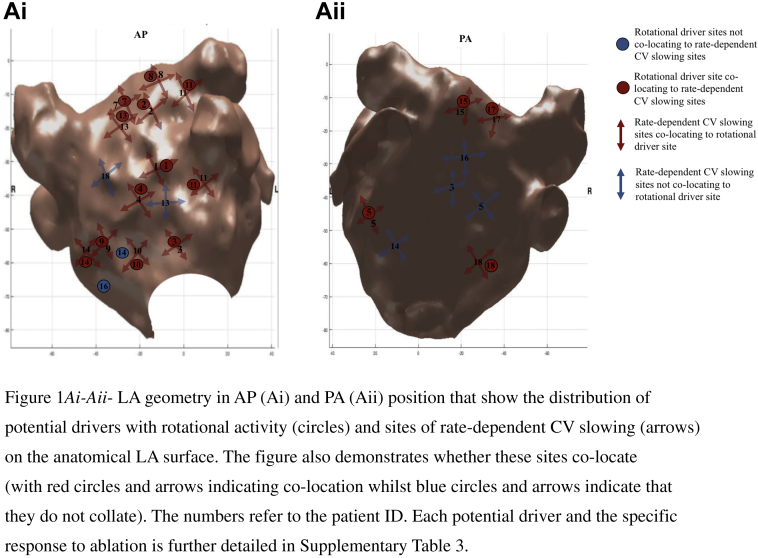
**Ai–Aii:** LA geometry in AP (**Ai**) and PA (**Aii**) position that show the distribution of potential drivers with rotational activity (*circles*) and sites of rate-dependent CV slowing (*arrows*) on the anatomical LA surface. The figure also demonstrates whether these sites co-locate (with *red circles* and *arrows* indicating co-location whilst *blue circles* and *arrows* indicate that they do not collate). The numbers refer to the patient ID. Each potential driver and the specific response to ablation is further detailed in Supplementary Table 3.

Eighteen of the 25 confirmed drivers (72.0%) were found at rate-dependent CV slowing sites (Supplemental Figure 1 and Supplemental Table 3), which included 16 of the 17 confirmed drivers with rotational activation (94.1%) but only 2 of 8 (25.0%) with focal activation (*P* = .001). Notably, 15 of the 18 (83.3%) confirmed drivers that resulted in AF termination were mapped to rate-dependent CV slowing sites (Figure 4).

### Potential predictive factors of confirmed driver sites

LVZs predicted driver sites with sensitivity of 72.0% (95% confidence interval [CI] 50.4%–87.1%) and specificity of 43.4% (95% CI 40.0%–46.9%).

Rate-dependent CV slowing sites showed sensitivity and specificity of 72.0% (95% CI 50.6%–87.9%) and 78.1% (95% CI 75.0%–80.9%), respectively, for predicting confirmed driver sites in AF, and sensitivity and specificity of 94.1% (95% CI 71.3%–99.9%) and 77.9% (95% CI 74.9%–80.7%), respectively, for predicting drivers with rotational activity. When only the 18 confirmed drivers mapped to LVZs were included, rate-dependent CV slowing sites showed sensitivity and specificity of 83.3% (95% CI 57.7%–95.6%) and 83.7% (95% CI 81.0%–86.2%), respectively, for predicting AF driver sites. Table 2 shows the value of different factors in predicting AF drivers mapped to LVZs.

**Table 2:** Value of different factors in predicting drivers in AF

Each LVZ island	AUC	*P* value	95% CI	Optimal cutoff value	Sensitivity	Specificity
Surface area (cm^2^)	0.63	.13	0.47–0.79	3.0	0.68	0.67
Mean bipolar voltage (mV)	0.81	<.001	0.68–0.93	0.28	0.88	0.63
SD of mean bipolar voltage (mV)	0.46	.62	0.29–0.63	0.10	0.56	0.50
CV at 600 ms (m/s)	0.76	.002	0.62–0.90	1.17	0.76	0.71
% CV measurements demonstrating rate-dependent CV slowing	0.86	<.001	0.73–0.98	19.1	0.80	0.91

AF = atrial fibrillation; AUC = area under the curve; CI = confidence interval; CV = conduction velocity; LVZ = low-voltage zone.

## Discussion

This is the first study to assess the interaction between CV dynamics and bipolar voltage in the human LA. A direct correlation between CV and bipolar voltage was observed. However, CV dynamics were not determined entirely by the presence or absence of LVZs, and not all LVZs were associated with rate-dependent CV slowing. Although CV was slowest in vLVZs (0–0.2 mV), these zones did not exhibit rate-dependent CV slowing. Sites of rate-dependent CV slowing predicted the sites of rotational activity in AF with high sensitivity and specificity, supporting the hypothesis that the rate-dependency of CV dynamics is mechanistically important in the development of reentrant arrhythmias.

### CV and bipolar voltage

LVZ were clustered in relatively large regions rather than scattered throughout the myocardium. The focal nature of this remodeling process has been observed previously^9^ and enabled accurate assessment of CV dynamics within these zones. CV at 600 ms PI was reduced by about 15% in LVZ (0.2–0.5 mV) and 40% in vLVZs (0–0.2 mV) compared to non-LVZs ≥0.5 mV, suggesting that CV slows progressively with fibrosis. The strong correlation between mean CV and both mean bipolar voltage and proportion of non-LVZs demonstrates a strong link between structural and electrical remodeling.

### CV dynamics and bipolar voltage

CV dynamics curves alter with structural remodeling,^4^ which was consistent with the study findings. With structural remodeling there is replacement of myocardial tissue by fibrosis,^6^ alteration in gap junction communication,^11^ and coupling of myocytes with fibroblasts.^12^ These phenomena may contribute to the slowing of conduction and altered CV dynamics seen in LVZs.

Atrial CV dynamics curves have not previously been studied in relation to structural remodeling. Patients with persistent AF have been shown to have broader CV dynamics curves than those with paroxysmal AF.^5^, ^13^ The finding that areas of structural remodeling have broad CV dynamics curves may explain this finding because atria in patients with persistent AF are often more dilated and scarred than in those with paroxysmal AF.^14^

### Relationship between rate-dependent CV slowing and drivers

Rate-dependent CV slowing sites were predominantly located in LVZs, and their prevalence increased with the proportion of the atrium made up by LVZs. Interestingly, they were limited to LVZs (0.2–0.5 mV). This is probably because this tissue is healthy enough to be capable of near-normal CV at longer PIs (600 ms) but is abnormal enough to reduce CV significantly with shorter PIs (<600 ms). In vLVZs (0–0.2 mV), tissue is likely to be markedly diseased, as reflected by the already very slow CV at 600 ms PI. This then shows little further slowing in CV at shorter PIs (ie, there is no conduction reserve).

Rate-dependent CV slowing sites were also seen in non-LVZs. Why certain areas with no fibrosis/scar showed enhanced CV slowing with rate is unclear. It is possible that sites defined as non-LVZs are not truly structurally normal, and there is the presence of subendocardial or epicardial fibrosis that would not be identified on endocardial bipolar voltage maps. This could account for the heterogeneity seen in apparently healthy tissue. However, these sites did not correlate to the location of reentry causing rotational activity in AF and therefore may not play an important mechanistic role. It may be that structural remodeling must be transmural to effectively promote reentry.

In this study, the majority of drivers had a rotational activation (62.0%), which is supported by the findings of our previous work.^7^ It has been suggested that localized slow conduction is necessary to maintain rotors, or at least that rotors may become anchored to such areas.^15^ In this study, 15 of the 17 confirmed drivers with rotational activation pattern were located in LVZs (0.2–0.5 mV) with rate-dependent CV slowing. The 11 focal activation patterns showed no predilection to LVZs and rate-dependent CV slowing sites, which is consistent with previous findings.^16^ It has been shown that sites with a broad CV dynamics curve have an alteration in activation vector and arcing with accelerated rates, which may reflect rate-dependent conduction block in certain directions.^5^ This may promote reentry.^17^ These data suggest that sites with a broad CV dynamics curve and enhanced rate-dependent CV slowing play an important role in the initiation and/or maintenance of reentrant mechanisms supporting AF. It may also explain why ablation of such areas has an organizing effect without simply creating an area of fixed conduction block to which reentry might anchor. It may be that ablation reduces the heterogeneity of CV by eliminating areas of rate-dependent CV slowing without the need to necessarily produce areas of continuous conduction block.

To our knowledge, CV restitution in the human atrium and its relationship to drivers mapped in AF have not previously been assessed. However, animal studies and computer modeling data investigating ventricular fibrillation have shown that rotors are associated with a broad CV restitution curve.^18^ This is consistent with the study findings whereby rotational drivers mapped to areas with a broad restitution curve and rate-dependent CV slowing.

The prevalence of rate-dependent CV slowing sites in LVZs may explain the poorer short- and long-term outcomes of AF catheter ablation in patients with more diseased atria.^19^ The study findings do point toward a potential role for a substrate modification approach to AF ablation based on LVZs and CV dynamics, because sites of rate-dependent CV slowing correlate to AF driver sites. Substrate modification based on voltage has shown promising results.^2^, ^3^ These data suggest that the targeting of scar could potentially be refined based on electrophysiological criteria.

Animal studies have elegantly shown that rotors can be functional and occur in normal tissue. We have previously demonstrated that drivers with rotational activity show a predilection for LVZs and that the amount of LVZ is predictive of the identification of rotational drivers.^20^ Others have demonstrated this independently using separate technologies.^21^ Computer modeling has suggested that fibrosis can anchor a rotor.^22^ It has also been shown that CV heterogeneity can promote reentry.^4^ Therefore, it is possible that rotors and other reentrant mechanisms might form within or gravitate toward LVZs, particularly those with heterogeneous conduction due to rate-dependent CV slowing.

### Study limitations

One of the main limitations of this study is the small patient numbers. This was overcome to some extent by assessing CV between more than a total of 3000 electrode pairs to allow regional analysis of multiple LVZs. The LA coverage achieved with the basket catheter is limited by catheter design and LA geometry, so the number of rate-dependent CV slowing sites may be underestimated. In this study, wavefront collision was not seen between electrode pairs over which CV was measured, but clearly this would impact CV measurements. Studies evaluating CV should consider this as a potential source of error and adjust the pacing site if needed.

The impact of fiber orientation and anisotropic effect on CV was not directly assessed in this study. However, sites of rate-dependent CV slowing were spatially stable and were not impacted by pacing site or the direction of wavefront propagation and thus are unlikely to be explained by anisotropy.

The aim of the study was to map the LA for possible AF drivers and assess for CV heterogeneity. Right atrial (RA) mapping with the CARTOFINDER system has not demonstrated RA drivers,^7^ so the RA was not mapped in this study. Therefore, whether this relationship between CV dynamics and drivers exists in the RA is uncertain.

What recording duration is optimal for the identification of potential drivers in AF remains unclear. It is possible that a longer recording may have identified more drivers. The CARTOFINDER mapping system uses a 30-second recording similar to that used by other mapping systems.^23^, ^24^ However, the drivers identified were observed repeatedly, and the response to ablation suggested they were mechanistically important. Indeed, drivers that demonstrate greater temporal stability may be more important in maintaining AF.^20^ The relevance of focal or rotational activity that occurs less frequently remains unclear.

In this study, we focused on electrophysiological endpoints to determine the mechanistic significance of potential drivers because there is arguably no other way to verify that a driver has been mapped. Other investigators have used termination of AF or cycle length prolongation as a surrogate for the interruption of mechanisms important for the maintenance of AF.^25^, ^26^ Larger multicenter studies powered to assess outcomes are currently in progress using CARTOFINDER to target drivers in AF (Clinicaltrials.gov Identifier: NCT03064451).

The voltage ranges studied were those conventionally regarded as LVZ, but it is accepted that areas with voltage >0.5 mV could still be abnormal. Further studies to define the lower limits of the normal range are desirable.

The current study used the ablation response to confirm the presence of drivers so as to correlate this with sites of CV heterogeneity. Further studies are needed to determine the impact of an approach targeting ablation based on local CV dynamics.

## Conclusion

Structural remodeling results in heterogeneous CV dynamics, which are determined largely by the degree of atrial disease. Moderately diseased tissue (0.2–0.5 mV) was most likely to display rate-dependent CV slowing, which showed a good correlation with rotational activation in AF. Notably, not all areas with voltage <0.5 mV seemed mechanistically important in this way. These data provide a potential rationale for randomized studies comparing long-term outcomes of an electrophysiological approach to substrate modification for AF based on CV dynamics to other ablation strategies.
