# Supplementary material for: Structural remodeling and conduction velocity dynamics in the human left atrium: Relationship with reentrant mechanisms sustaining atrial fibrillation
Source: Heart Rhythm. 2019 Jan;16(1):18–25. doi: 10.1016/j.hrthm.2018.07.019 (PMC6317307; doi:10.1016/j.hrthm.2018.07.019)
Supplement: Supplemental Methods [file mmc1.docx]

SUPPLEMENTAL METHOD

*i) CARTOFINDER mapping system*

Is a novel mapping system which allows the creation of global dynamic wavefront maps using local activation time obtained from the individual electrode poles of a whole-chamber basket catheter. The local activation times are obtained through annotation at the peak negative dv/dt on atrial unipolar signals recorded on the basket catheter whilst referencing to Wilson’s central terminal. To avoid inappropriate annotation on far-field ventricular signals these are initially filtered out. To then ensure annotation of true atrial signal and avoiding the annotation of sites of fractionation or noise, the CARTOFINDER system creates a bipolar electrogram window whereby each electrode is paired with its 2 nearest electrodes (one ahead and one behind). Only signals that fall within this window are annotated excluding signals outside this window that can represent fractionation and noise. Electrogram annotation can be reviewed and edited in an open format. The timing of electrodes are then displayed on the geometry relative to each other for a 250ms window that then moves through the 30-seconds recording in a dynamic fashion.

*ii) Bipolar voltage map*

Points that were ≥3mm from the geometry surface were filtered as not being in contact with the myocardium, and points were respiratory gated. Points were taken in sinus rhythm. An interpolation threshold of 5mm was used for surface color projection and points were collected aiming for complete coverage (i.e. no areas >5mm from a data point). Bipolar electrograms were filtered at 30 to 500Hz and the peak-to-peak amplitude was recorded on CARTO. Bipolar voltages obtained at the pulmonary veins (PVs) were excluded to allow for a mean bipolar voltage of the LA body only.

*iii) Ablation strategy*

In brief all patients underwent pulmonary vein (PV) isolation with additional ablation targeting drivers identified on post-PV isolation maps. A lesion was delivered at the center of the driver site with further ablation surrounding the initial lesion in a cluster, avoiding the creation of linear lesions. Ablation was stopped once the study pre-defined ablation response was achieved, no residual signal identified at the ablation site or >5 minutes of ablation had been performed.

*iv) Local CVs*

Electrogram waveforms, data point location and 3D left atrial geometry data were imported into MATLAB (MathWorks, MA) and utilizing a custom written script each basket catheter electrode was paired to a neighboring electrode within a known geodesic distance ensuring the electrodes were mapping the same anatomical surface. Pairs lacking geometry contact were excluded from the analysis only leaving pairs with adequate contact. Contact was defined as per previous study (10) whereby electrodes <10mm from the geometry were defined as being in contact. The electrograms were then reviewed on electrodes that were within 10mm of the geometry to ensure that electrograms were adequate for analysis. This thereby left pairs that were within a known geodesic distance and with adequate contact. Following this, the position of these pairs were verified on the geometry and reviewing the wavefront propagation maps created during pacing with CARTOFINDER only those electrode pairs that were orientated parallel to the direction of wavefront propagation were used for CV measurements. Determination of wavefront propagation with pacing using CARTOFINDER has previously been validated (11). This process was conducted for all four pacing sites and PIs.

To determine CV, firstly the local activation time was calculated as the interval between the pacing spike and steepest descent (peak negative dv/dt) in the unipolar electrogram. The last beat of the 30-second recording was used. The CV between each electrode pair was defined as the geodesic distance divided by the activation time difference and expressed in m/s. By using the geodesic distance to determine the distance the wavefront has travelled in 3D space (rather than simply the shortest distance between electrodes), and by only including activation patterns with the wavefront moving parallel to the orientation of the electrodes, this should allow accurate determination of conduction velocity rather than simply activation time as others have studied previously. Pairs with an activation time difference of <1ms at 600ms PI were excluded as sites of simultaneous activation. CV was assessed over a distance of 5-30mm with electrode pairs closer or further apart than this excluded from the analysis.

*v) CV and AF driver sites*

Arrhythmia was induced following the study protocol by burst atrial pacing from the CS starting at PI of 400ms, with a 10ms decrement until either arrhythmia was induced or reaching 200ms. If this did not induce the arrhythmia then this was repeated from elsewhere in the atria. Following AF induction, 10min were allowed for AF to stabilize.

AF CL was measured over 30 consecutive cycles from the PentaRay catheter positioned in the LA appendage before and after ablation.
