# Supplementary material for: Structural remodeling and conduction velocity dynamics in the human left atrium: Relationship with reentrant mechanisms sustaining atrial fibrillation
Source: Heart Rhythm. 2019 Jan;16(1):18–25. doi: 10.1016/j.hrthm.2018.07.019 (PMC6317307; doi:10.1016/j.hrthm.2018.07.019)
Supplement: Supplemental Table 1 [file mmc2.docx]

*Supplemental Table 1- Patient demographics*

| **Baseline characteristics** | | **Cohort n=18** |
| --- | --- | --- |
| Age yrs. mean ± SD | 62±10 | |
| Male n (%) | 10 (56) | |
| DM n (%) | 0 (0) | |
| Hypertension n (%) | 7 (39) | |
| CVA^Ţ^ n (%) | 1 (6) | |
| Ischaemic heart disease n (%) | 2 (11) | |
| Previous cardiac surgery n (%) | 1 (6) | |
| Left ventricular EF^Ŧ^≥ 55% n (%) | 15 (83) | |
| Left atrial area cm^2^  20-30  30-40 | 14 (78)  4 (22) | |
| AF duration months mean ± SD | 16.5±5.2 | |
| Medication  Amiodarone  Flecainide  Bisoprolol/Sotalol  Verapamil | 9 (50)  1 (6)  14 (78)  1 (6) | |
| RF ablation at a driver site min mean ± SD | 2.9±1.1 | |
| LA coverage achieved with basket catheter % mean ± SD | 74.1±7.3 | |
| Complications n | 0 | |

^*^CVA- Cerebrovascular accident

^Ţ^ EF- Ejection fraction
