# Supplementary material for: Structural remodeling and conduction velocity dynamics in the human left atrium: Relationship with reentrant mechanisms sustaining atrial fibrillation
Source: Heart Rhythm. 2019 Jan;16(1):18–25. doi: 10.1016/j.hrthm.2018.07.019 (PMC6317307; doi:10.1016/j.hrthm.2018.07.019)
Supplement: Supplemental Table 2 [file mmc3.docx]

*Supplemental Table 2- CV reduction with increasing pacing rates stratified by voltage.*

|  | Non-LVZs ≥0.50mV | LVZs [0.2-0.5mV] | vLVZs [0-0.2mV] | p-value |
| --- | --- | --- | --- | --- |
| Mean CV^Ţ^m/s at 600ms ± SD | 1.59±0.13 | 1.18±0.13 | 0.78±0.09 | <0.001 |
| Mean CV m/s change ± SD  600-450ms PIs^Ŧ^  450-300ms PIs  300-250ms PIs | 0.01±0.01  0.03±0.02  0.59±0.09 | 0.05±0.03  0.12±0.05  0.12±0.06 | 0.01±0.02  0.02±0.01  0.03±0.02 | <0.001  <0.001  <0.001 |
| % of RD^§^ CV slowing sites | 24.3±15.0 | 75.7±15.5 | 0 | <0.001 |

^Ţ^CV- conduction velocity

^Ŧ^PI- pacing interval

^§^RD- rate dependent
