# Supplementary material for: Structural remodeling and conduction velocity dynamics in the human left atrium: Relationship with reentrant mechanisms sustaining atrial fibrillation
Source: Heart Rhythm. 2019 Jan;16(1):18–25. doi: 10.1016/j.hrthm.2018.07.019 (PMC6317307; doi:10.1016/j.hrthm.2018.07.019)
Supplement: Supplemental Table 3 [file mmc4.docx]

*Supplemental Table 3- Shows the characteristics of potential AF drivers mapped with the CARTOFINDER system and correlation with rate-dependent CV slowing sites*

| Patient ID | Driver type | | Driver location  in LA |  | Correlation with rate-dependent CV slowing sites | Ablation Response | |  |
| --- | --- | --- | --- | --- | --- | --- | --- | --- |
| 1 | Focal  Rotational | Anterior LAA^Ţ^  Mid Anterior | | | No SCL  Yes AT | |  | |
| 2 | Rotational | Mid Roof | | | Yes Sinus | |  |  |
| 3 | Rotational | Low Anterior | | | Yes AT | |  |  |
| 4 | Focal  Rotational | Lateral  Mid Anterior | | | No SCL  Yes Sinus | |  |  |
| 5 | Focal  Rotational | Mid Anterior  Posterior/inferior lateral | | | No SCL  Yes AT | |  |  |
| 6 | Focal | Lateral | | | No AT | |  |  |
| 7 | Focal  Rotational | Low Anterior  Mid Roof | | | No SCL  Yes Sinus | |  |  |
| 8 | Rotational | Roof | | | Yes AT | |  |  |
| 9 | Rotational | Anteroseptal | | | Yes AT | |  |  |
| 10 | Rotational | Anteroseptal | | | Yes AT | |  |  |
| 11 | Rotational  Rotational | Mid Anterior  Roof/LAA | | | Yes SCL  Yes AT | |  |  |
| 12 | Focal | Anterior LAA | | | Yes Sinus | |  |  |
| 13 | Rotational | Mid Roof | | | Yes AT | |  |  |
| 14 | Focal  Rotational  Rotational | Lateral  Anteroseptal  Septal | | | No Nil  No Nil  Yes AT | |  |  |
| 15 | Rotational | Posterior roof | | | Yes AT | |  |  |
| 16 | Focal  Rotational  Focal | Anterior LAA  Septal  Lateral | | | No SCL  No SCL  Yes AT | |  |  |
| 17 | Focal  Rotational | Posterolateral  Posterior Roof | | | No Nil  Yes Sinus | |  |  |
| 18 | Focal  Rotational | Posterior/inferior  Posteroseptal | | | No Nil  Yes Sinus | |  |  |
|  |  |  | | |  | |  |  |

^Ţ^LAA- Left atrial appendage
